# Supplementary material for: Genome-wide profiling in colorectal cancer identifies PHF19 and TBC1D16 as oncogenic super enhancers
Source: Nat Commun. 2021 Nov 4;12:6407. doi: 10.1038/s41467-021-26600-5 (PMC8568941; doi:10.1038/s41467-021-26600-5)
Supplement: Supplementary file 1 — Supplementary files [file 41467_2021_26600_MOESM1_ESM.pdf]

Sup. Fig. S1

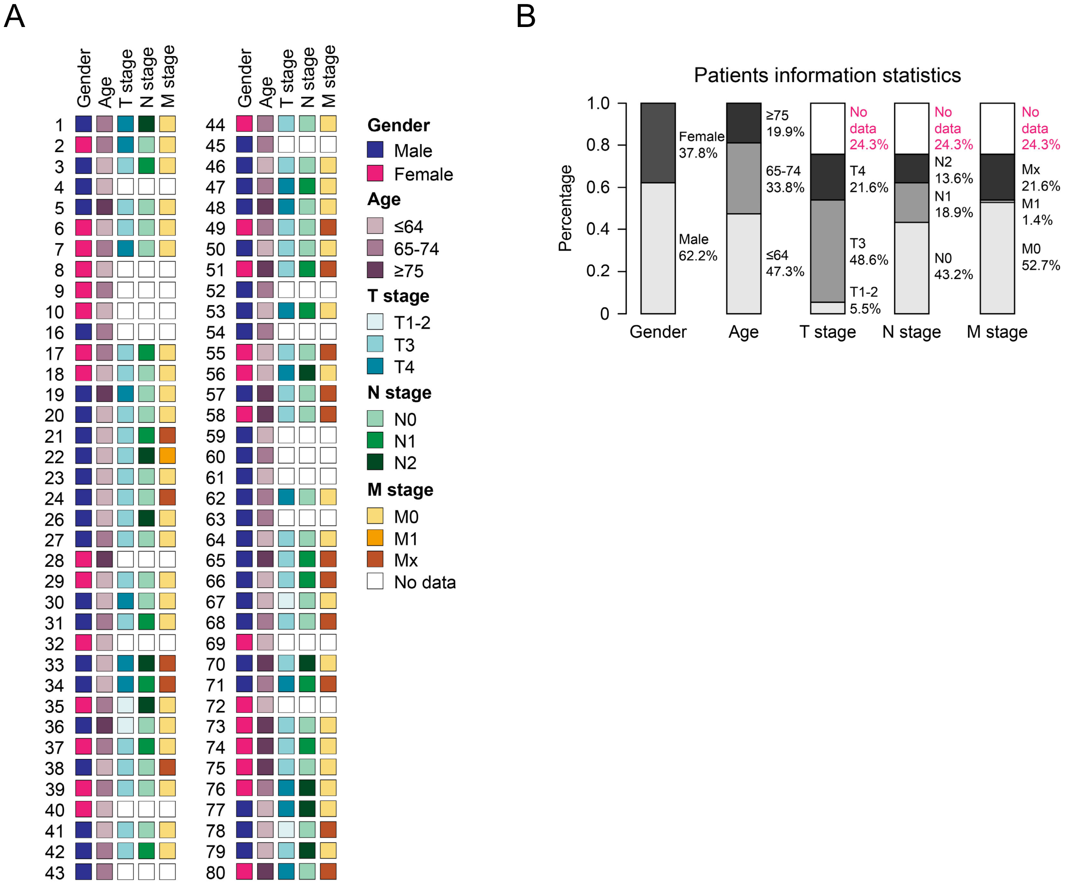

Sup. Fig. S1 Information of collected CRC tissues. (A) The clinical information of all 74 CRC patients participated in our study. **(B)** Barplot showing the clinical information statistics of CRC patients.

Sup. Fig. S2

A

Genomic element	Tissue	Total number
Promoter	Native	9896
Promoter	Tumor	10663
Enhancer	Native	27156
Enhancer	Tumor	39207

B

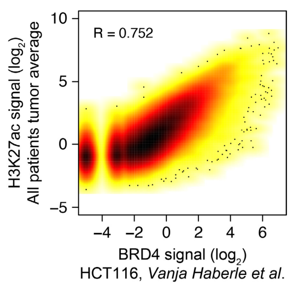

C

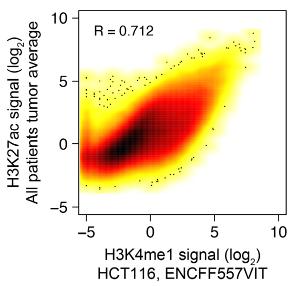

D

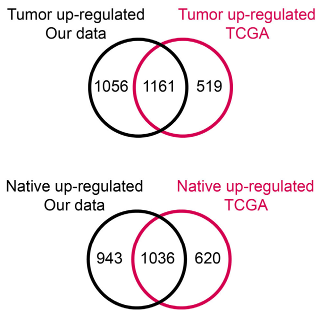

E

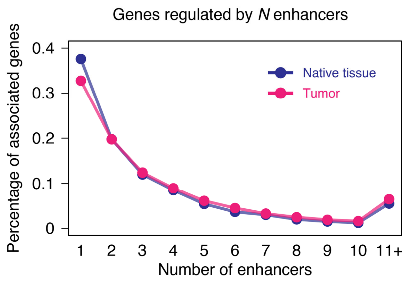

F

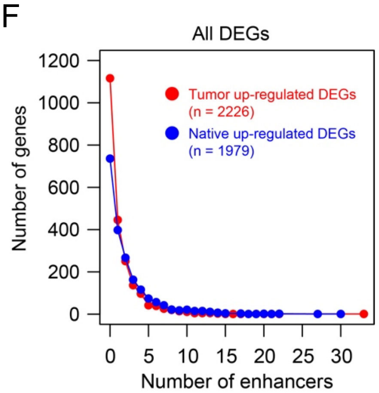

G

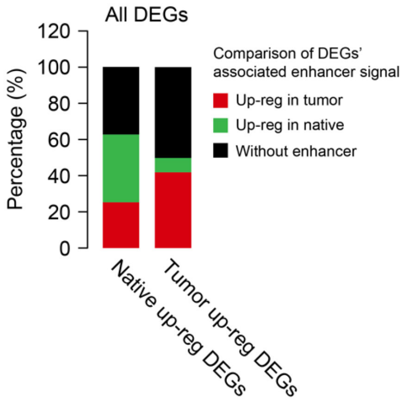

H

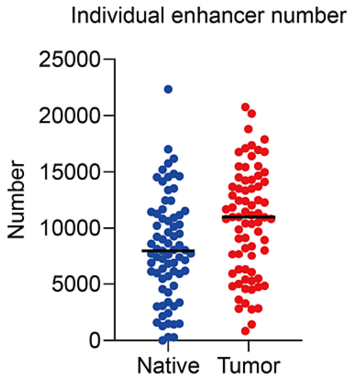

Enhancer number

	Native	Tumor
Minimum	13	838
Maximum	22339	20770
Mean	8198	10526
Median	7999	10980

I

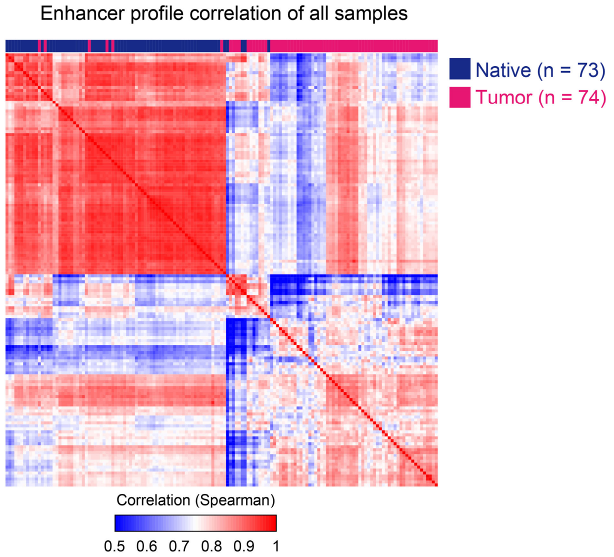

J

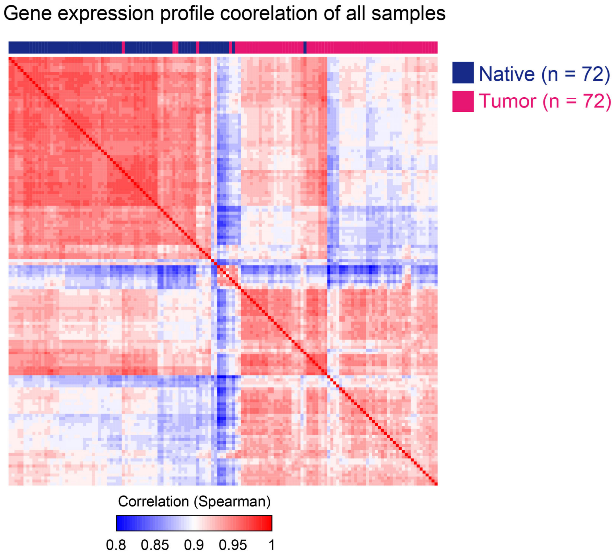

Sup. Fig. S2, continued

K

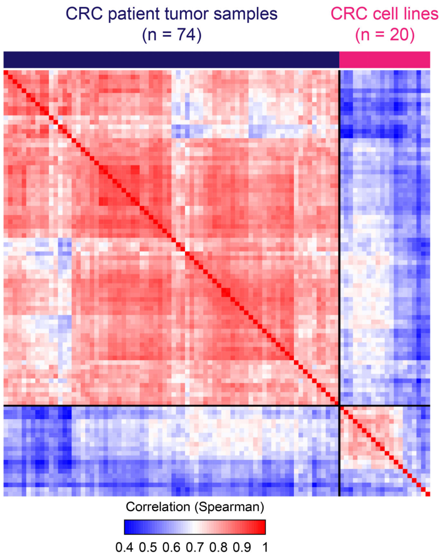

Sup. Fig. S2 The enhancer analysis of CRC samples. (A) The number of significant promoters and enhancers in tumor and native tissues. **(B-C)** Mean H3K27ac level in CRC tumors versus BRD4 (B) and H3K4me1 (C) levels in HCT116. **(D)** Overlap of tumor (top) and native (bottom) higher-expressed genes in our data with those in TCGA COAD data. **(E)** Proportion of enhancer/gene assignments to assigned enhancer number in tumor and native tissues. **(F)** Dot plot shows the relationship between the gene number and their associated enhancer number in both tumor and native tissue. **(G)** Bar plot for the proportion of DEGs with different enhancer signal alteration in tumor and native tissue. **(H)** Dot plot (left panel) and statistical values (right panel) of Enhancer number for all the CRC patients native and tumor samples. **(I)** Heatmap for the spearman correlation of enhance signal (H3K27ac RPM) among tumor and native tissue samples from all CRC patients. **(J)** Heatmap for the spearman correlation of gene expression profiles among tumor and native tissues from all CRC patients. **(K)** Heatmap for the spearman correlation of enhancer profiles among all CRC patient tumor samples and 20 CRC cell lines.

Sup. Fig. S3

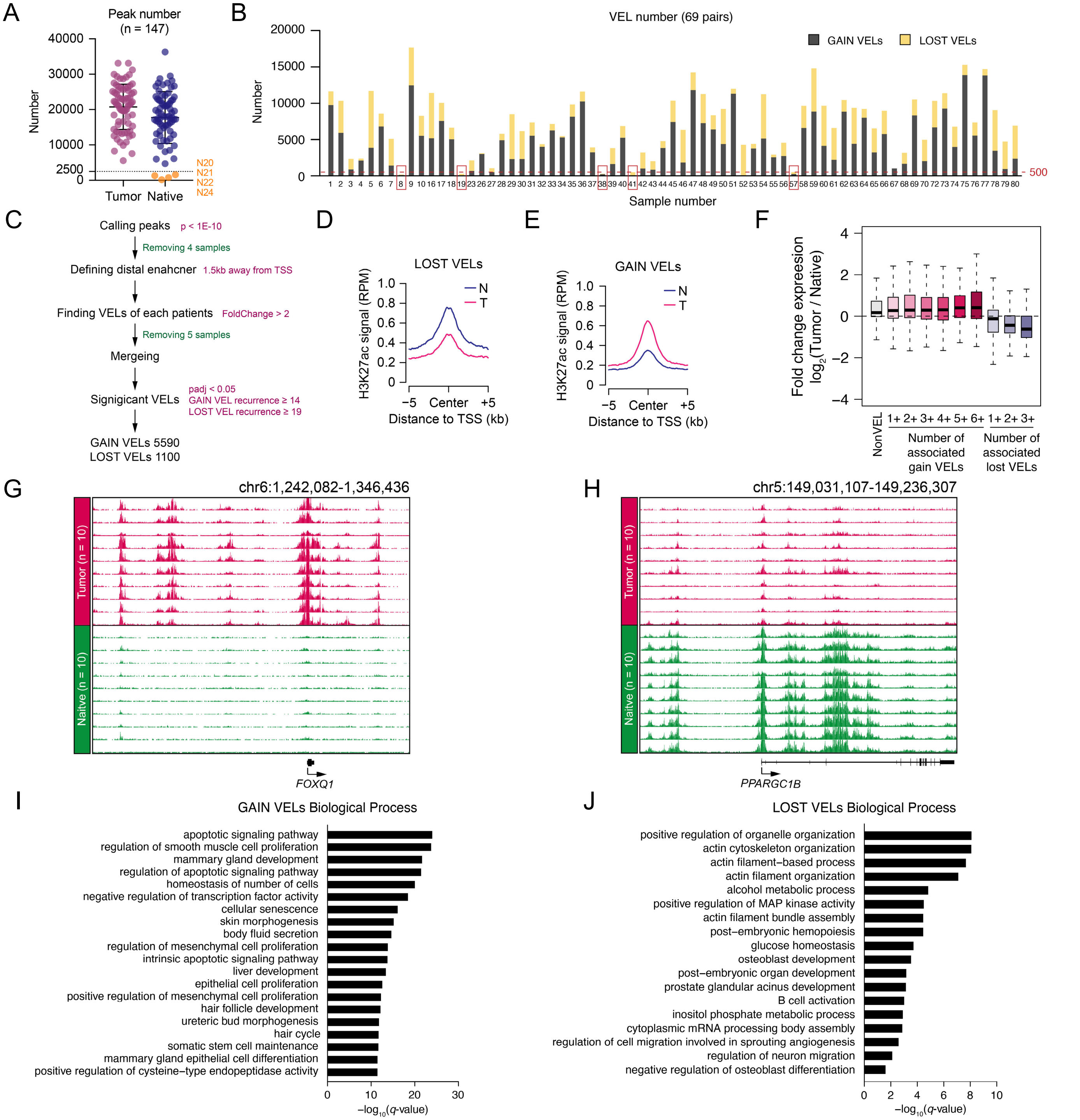

Sup. Fig. S3 Functional analysis of gain and lost enhancers in CRC. (A) The number of significant H3K27ac peaks in each sample. The samples whose peak number less than 2500 was highlighted in yellow. The top and bottom error bar indicate 75th and 25th percentile respectively. **(B)** VEL (including gain and lost VELs) number of each pair of samples. The patient whose VEL number less than 500 was highlighted in red box. **(C)** Flow chart of VEL definition. **(D&E)** The average H3K27ac signal (RPM) in the region of lost (D) and gain (E) VELs. **(F)** Fold change of gene expression associated with gain (red) and lost (purple) VELs, and not associated with VELs (nonVEL; grey). The box represent the data from 25th to 75th percentile, black line represent the median, and the top and bottom whiskers represent the 75th percentile + 1.5* interquartile range and 25th percentile + 1.5* interquartile range respectively. **(G)** Representative H3K27ac tracks of gain VEL in *FOXO1* gene loci. **(H)** Representative H3K27ac tracks of lost VEL in *PPARGC1B* gene loci. **(I&J)** The biological processes in which the genes associated with gain (J) and lost (K) VELs were enriched, analyzed by GREAT (version 3.0.0).

Sup. Fig. S4

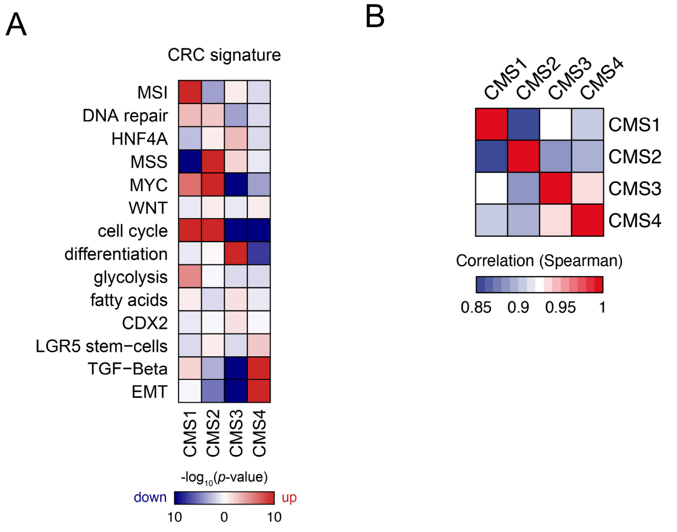

Sup. Fig. S4 The consensus molecular subtypes (CMS) classification of CRC samples. (A) The significance of CRC signature for four CMS subgroups identified by CMScaller. Two-sided test is utilized here. **(B)** Correlation of mean H3K27ac on the regions of gain VELs in four CMS subgroups. Correlations were calculated by Spearman correlation coefficient.

Sup. Fig. S5

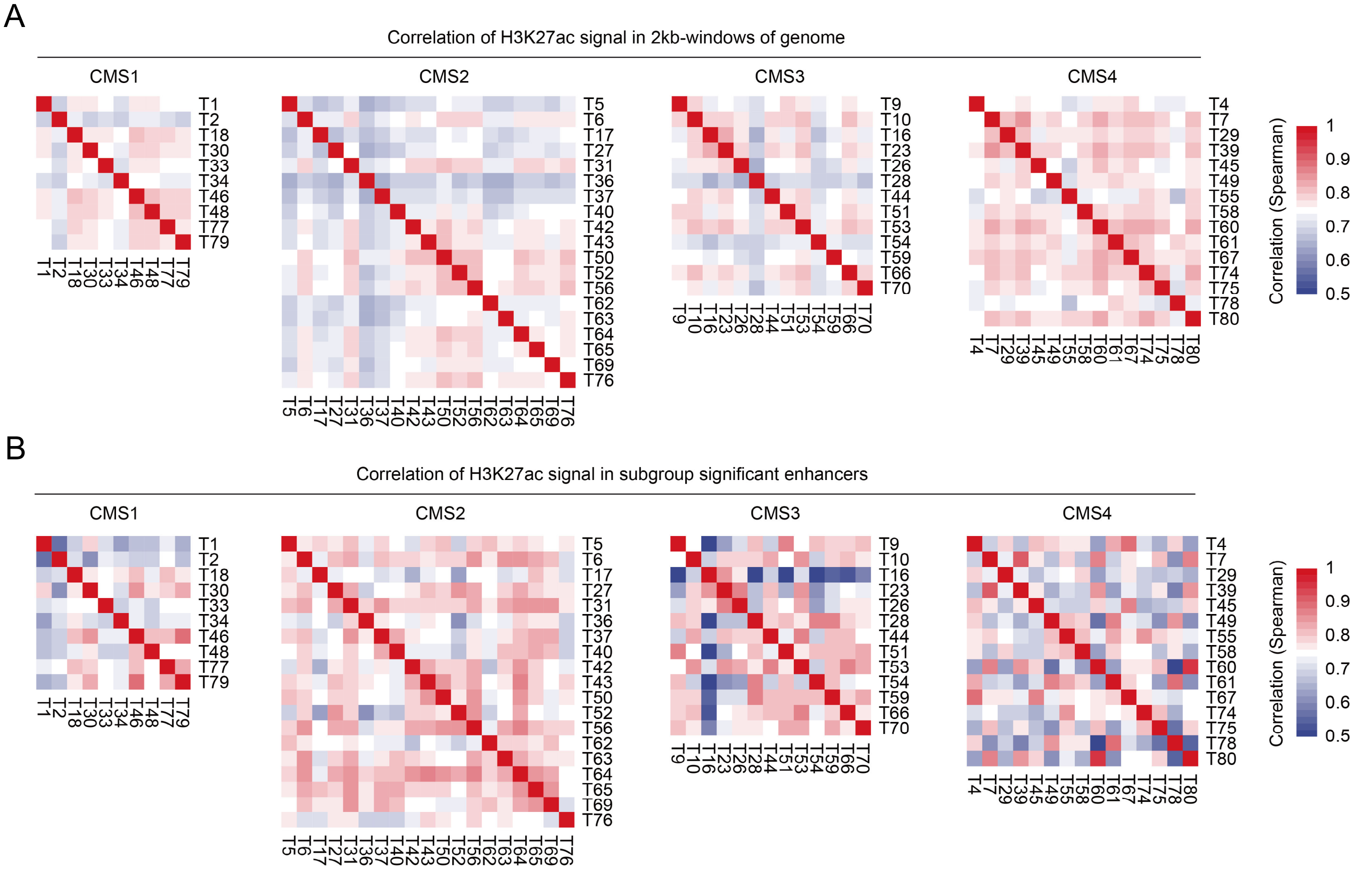

Sup. Fig. S5 Correlation of H3K27ac in four CMS subgroups. (A) H3K27ac RPM values were generated in each 2-kb window for the entire genome and compared between different samples for correlation analysis (Spearman). Correlation of H3K27ac signal at 2kb-windows of genome in all tumor samples from CMS1-4 subgroup. **(B)** Heatmap showing the correlation of H3K27ac signal at CMS-specific gain VELs in tumor samples of each subgroup. Correlations were calculated by Spearman correlation coefficient.

Sup. Fig. S6

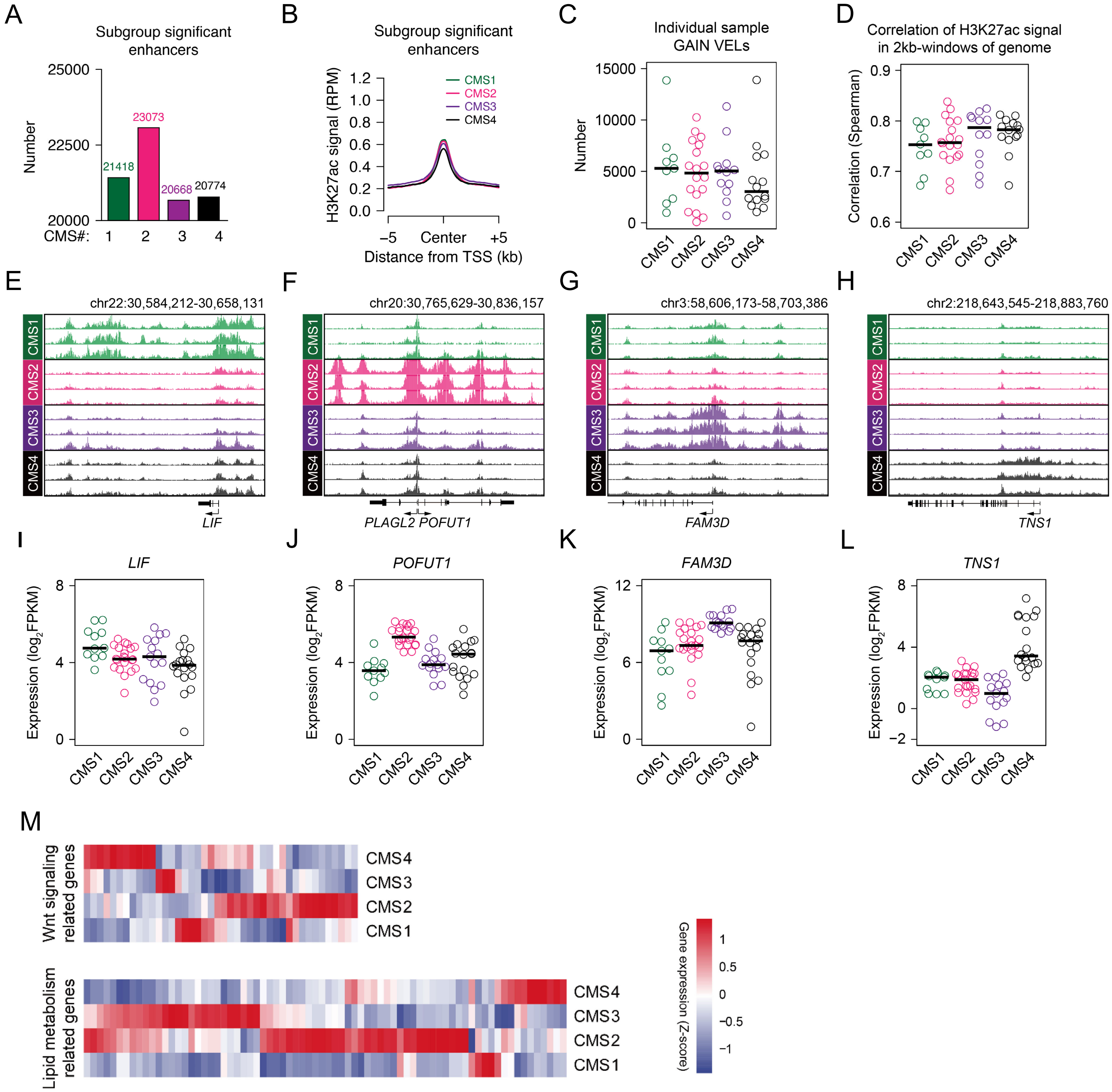

Sup. Fig. S6 The enhancer analysis of four CMS groups. (A) The number of significant enhancers in four CMS subgroups. **(B)** The average H3K27ac signal (RPM) at the regions of specific significant enhancers in four CMS subgroups. **(C)** The number of gain VELs of individual samples of each subgroup. Black lines indicate the median number. The sample number is CMS1 = 9, CMS2 = 18, CMS3 = 12 and CMS4 = 14. **(D)** Correlation of H3K27ac signal at 2kb-windows of genome between paired tumor and native tissues in four CMS subgroups. Correlations were calculated by Spearman correlation coefficient. Black lines indicate the median number. The samples number is the same as Fig. S6C. **(E-H)** Normalized H3K27ac tracks for representative gain VEL samples of four CMS subgroups. **(I-L)** Expression of the above four genes in S6E-H in tumor samples of four subgroups. Black lines indicate the median. The samples number is the same as Fig. S6C. **(M)** Heatmaps to show the relative expression level of genes related with lipid metabolism and Wnt signaling.

Sup. Fig. S7

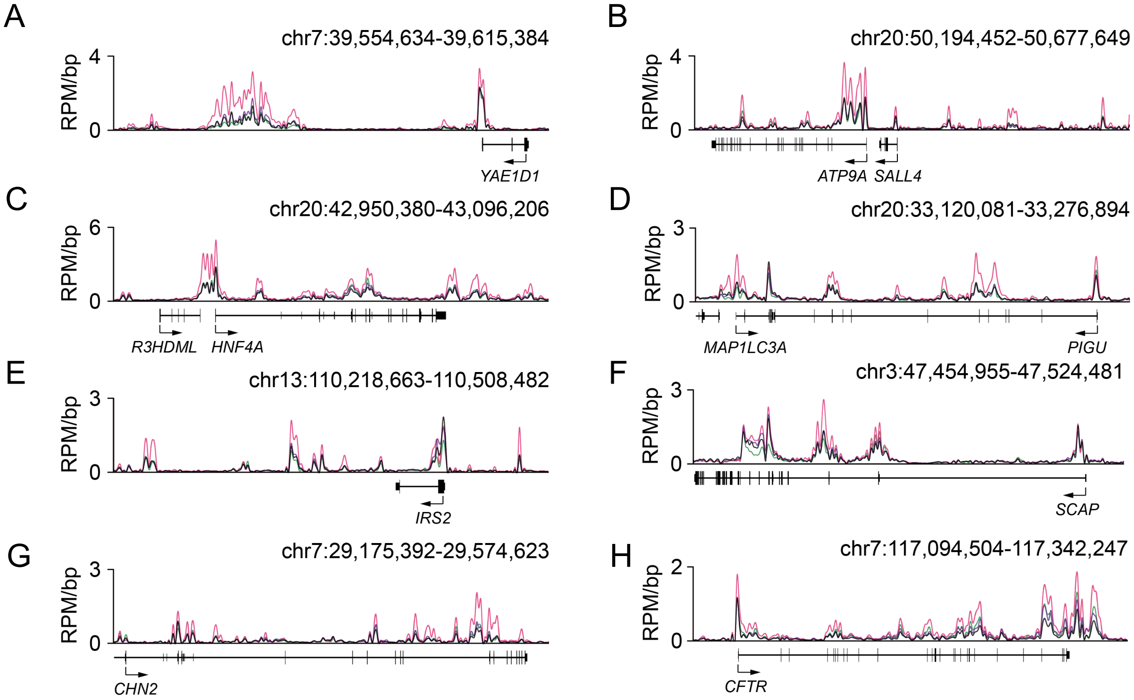

Sup. Fig. S7 H3K27ac tracks of representative genes in four CMS groups. (A-H) Meta normalized H3K27ac tracks on *YAE1D1* (A), *ATP9A* (B), *HNF4A* (C), *PIGU* (D), *IRS2* (E), *SCAP* (F), *CHN2* (G) and *CFTR* (H) loci in four CMS subgroups.

Sup. Fig. S8

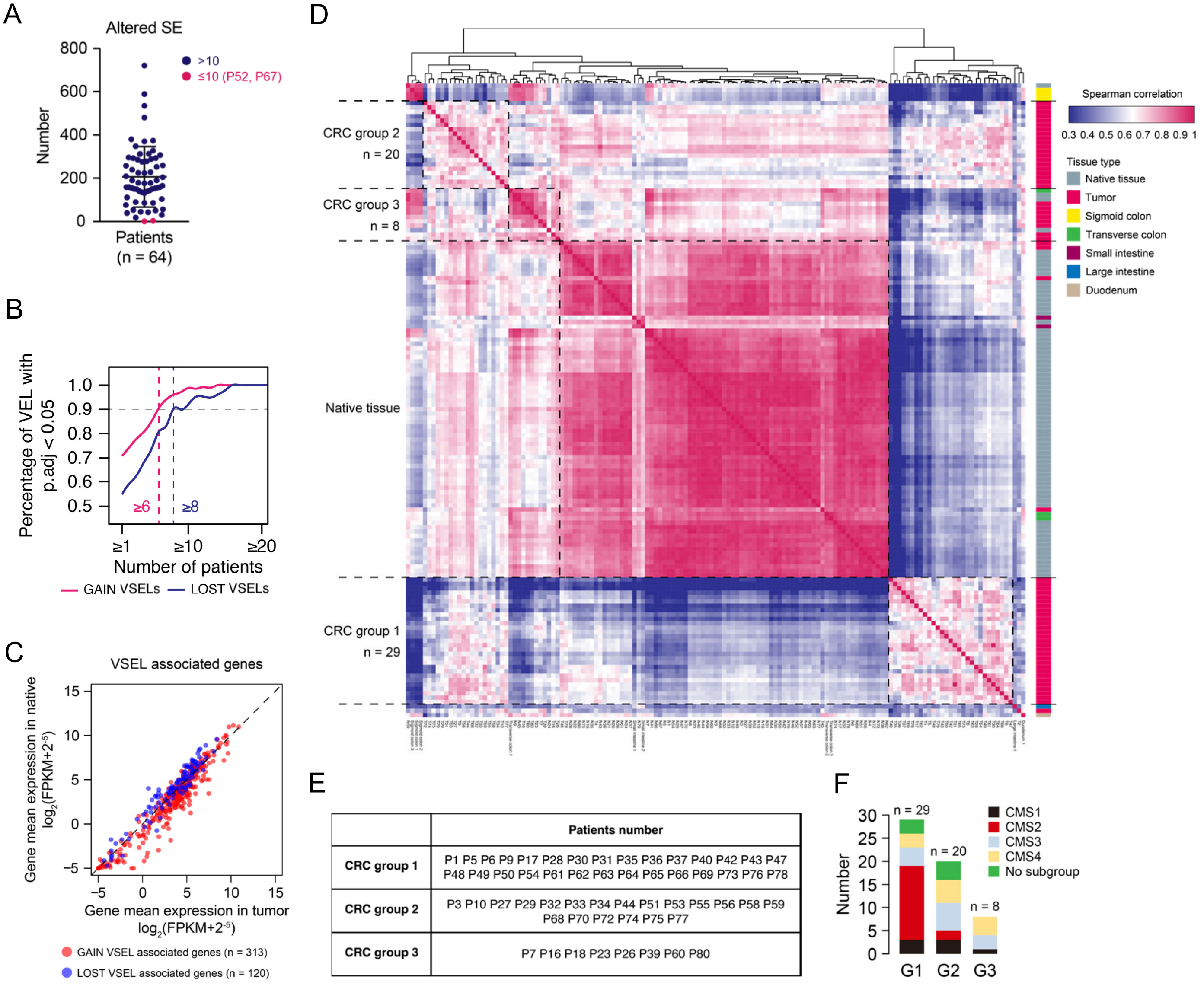

	Patients number
CRC group 1	P1 P5 P6 P9 P17 P28 P30 P31 P35 P36 P37 P40 P42 P43 P47 P48 P49 P50 P54 P61 P62 P63 P64 P65 P66 P69 P73 P76 P78
CRC group 2	P3 P10 P27 P29 P32 P33 P34 P44 P51 P53 P55 P56 P58 P59 P68 P70 P72 P74 P75 P77
CRC group 3	P7 P16 P18 P23 P26 P39 P60 P80

Sup. Fig. S8 Analysis of super enhancers (SE) in CRC. (A) The number of significant VSELs (FC > 2) in each sample. The pairs whose VSEL number less than 10 were highlighted in red. The top and bottom error bar indicate 75th and 25th percentile respectively. **(B)** The recurrence requirements for VSELs to meet statistical significance (p.adj < 0.05). The two vertical dashed lines highlight the recurrence of gain (red) and lost (blue) VSELs when achieving the cut-off (0.9, black horizontal dashed line) of significant percentage. For gain and lost VSELs, the significant percentage are 94.098% and 90.298% when reach to the cut-off. p.adj indicates the BH adjusted t-test p-value. Two-sided test utilized here. **(C)** Scatter plot showing the mean gene expression of gain and lost VSEL-associated genes in tumor and native tissues. **(D)** Heatmap to visualize the unsupervised clustering of pairwise correlations of H3K27ac signal (RPM) on VSELs (334 gain VSELs and 121 lost VSELs) for all. H3K27ac ChIP-seq data of five normal intestinal tissues downloaded from ENCODE were used, sigmoid colon (ENCFF611MTD, ENCFF860KII and ENCFF250TIL), transverse colon (ENCFF639EGR, ENCFF538APS and ENCFF485QGB), small intestine (ENCFF195YMX and ENCFF943RFS), large intestine (ENCFF805JFJ) and duodenum (ENCFF630DOD). **(E)** The members of CRC patient groups indicated in Sup. 4C. **(F)** Bar plot showing the overlap between our cluster analysis identified samples and CMS subgroup samples.

Sup. Fig. S9

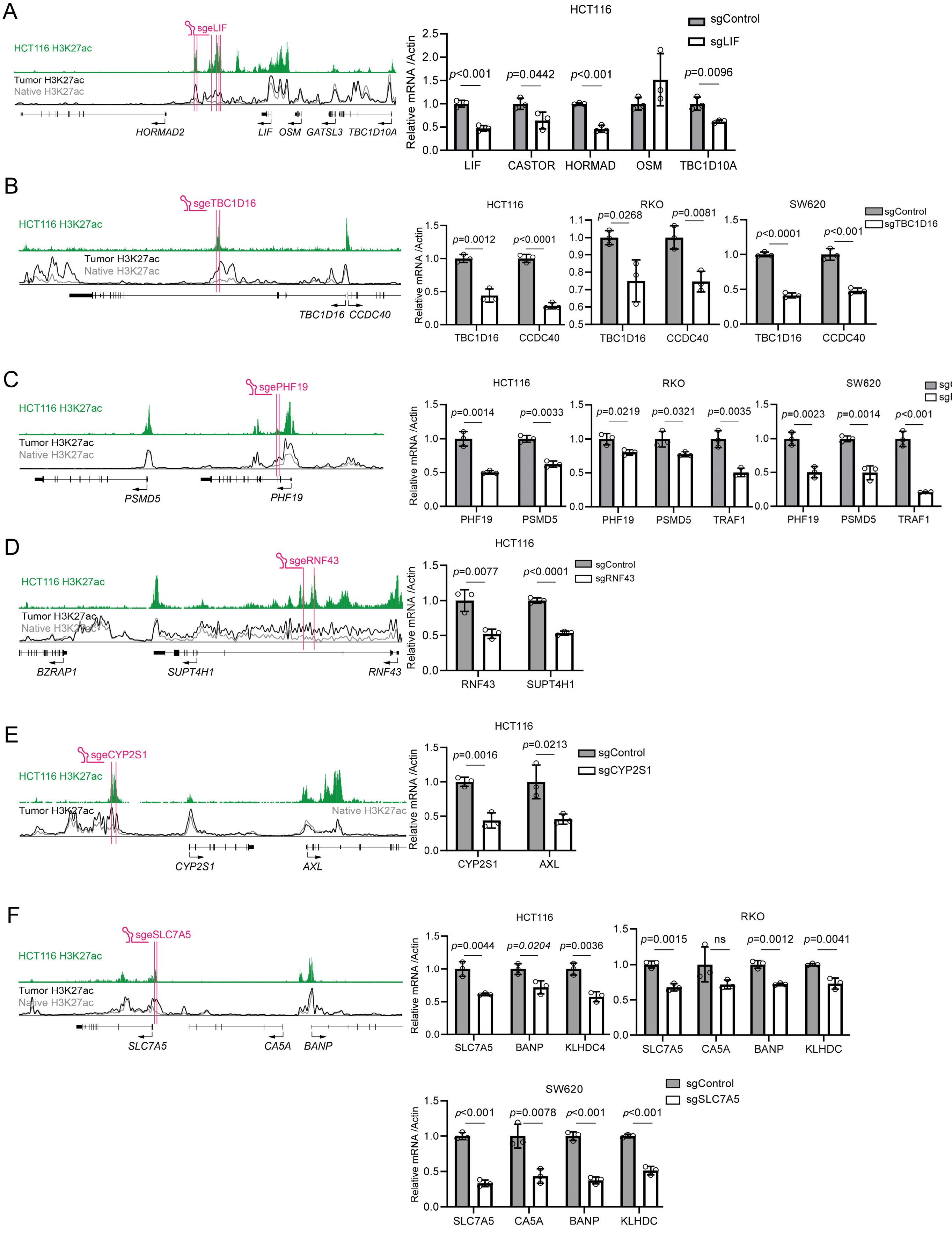

A
sgeLIF
HCT116 H3K27ac
Tumor H3K27ac
Native H3K27ac
HORMAD2
LIF
OSM
GATSL3
TBC1D10A
HCT116
sgControl
sgLIF
Relative mRNA /Actin
p<0.001
p=0.0442
p<0.001
p=0.0096
LIF
CASTOR
HORMAD
OSM
TBC1D10A
B
sgeTBC1D16
HCT116 H3K27ac
Tumor H3K27ac
Native H3K27ac
TBC1D16
CCDC40
HCT116
RKO
SW620
sgControl
sgTBC1D16
p=0.0012
p<0.0001
p=0.0268
p=0.0081
p<0.0001
p<0.001
TBC1D16
CCDC40
C
sgePHF19
HCT116 H3K27ac
Tumor H3K27ac
Native H3K27ac
PSMD5
PHF19
HCT116
RKO
SW620
sgControl
sgPHF19
p=0.0014
p=0.0033
p=0.0219
p=0.0321
p=0.0035
p=0.0023
p=0.0014
p<0.001
PHF19
PSMD5
TRAF1
D
sgeRNF43
HCT116 H3K27ac
Tumor H3K27ac
Native H3K27ac
BZRAP1
SUPT4H1
RNF43
HCT116
sgControl
sgRNF43
p=0.0077
p<0.0001
RNF43
SUPT4H1
E
sgeCYP2S1
HCT116 H3K27ac
Tumor H3K27ac
Native H3K27ac
CYP2S1
AXL
HCT116
sgControl
sgCYP2S1
p=0.0016
p=0.0213
F
sgeSLC7A5
HCT116 H3K27ac
Tumor H3K27ac
Native H3K27ac
SLC7A5
CA5A
BANP
HCT116
RKO
SW620
p=0.0044
p=0.0204
p=0.0036
p=0.0015
ns
p=0.0012
p=0.0041
p<0.001
p=0.0078
p<0.001
p<0.001
SLC7A5
BANP
KLHDC4
CA5A
KLHDC
sgControl
sgSLC7A5

Sup. Fig. S9, continued

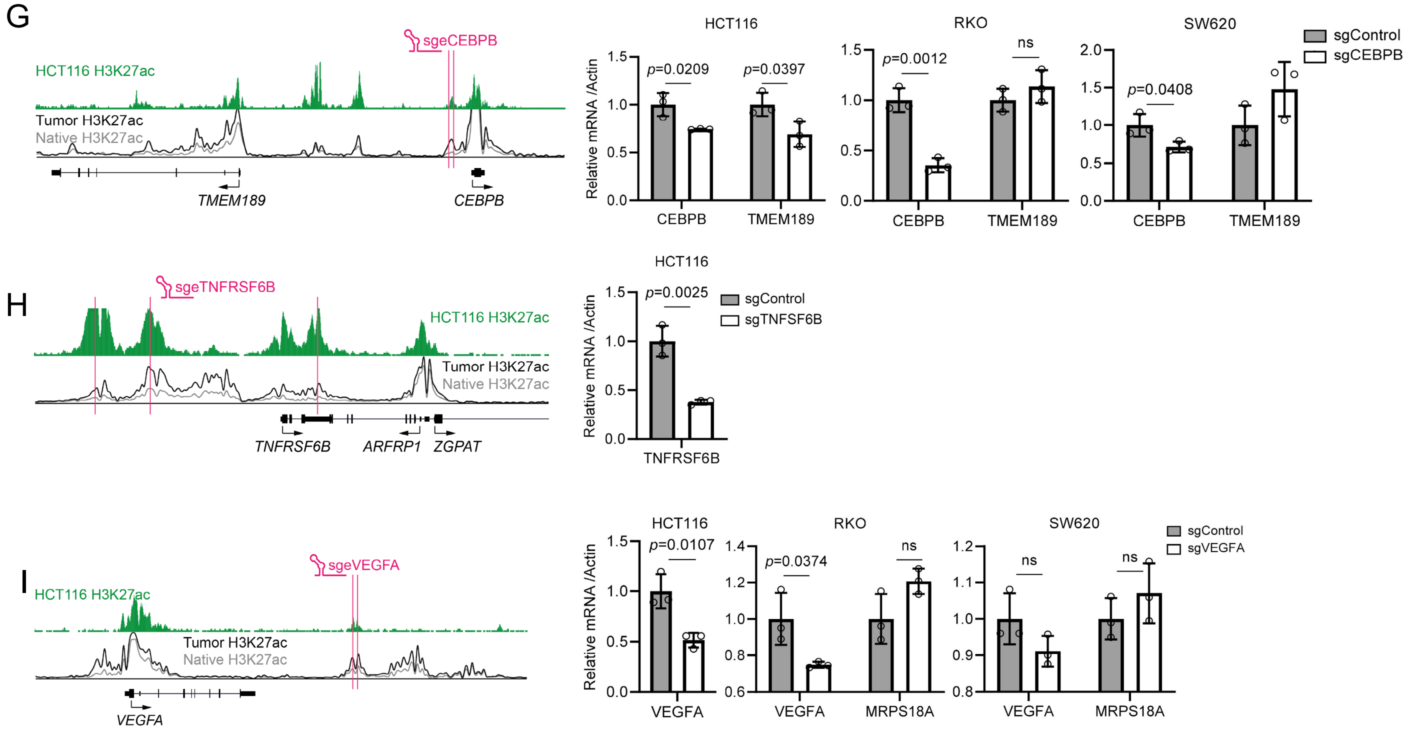

Sup. Fig. S9 Experimentally verification of CRC specific super enhancers. (A-I) The design of sgRNA targeting SEs were shown on the left, and their effects on gene transcription determined by quantitative PCR were shown on the right. The pink lines represent the target genome site for sgRNAs. Cell were transfected with multiple sgRNAs together and the antibiotic-selected pooled stable cells were used for study. Multiple cell lines were used for the SEs functioning in other assays. n = 3 for all samples. Data are presented as mean values +/- SEM. Statistical analysis was performed using a two-sided Student t test. p value was labelled on the corresponding items.

Sup. Fig. S10

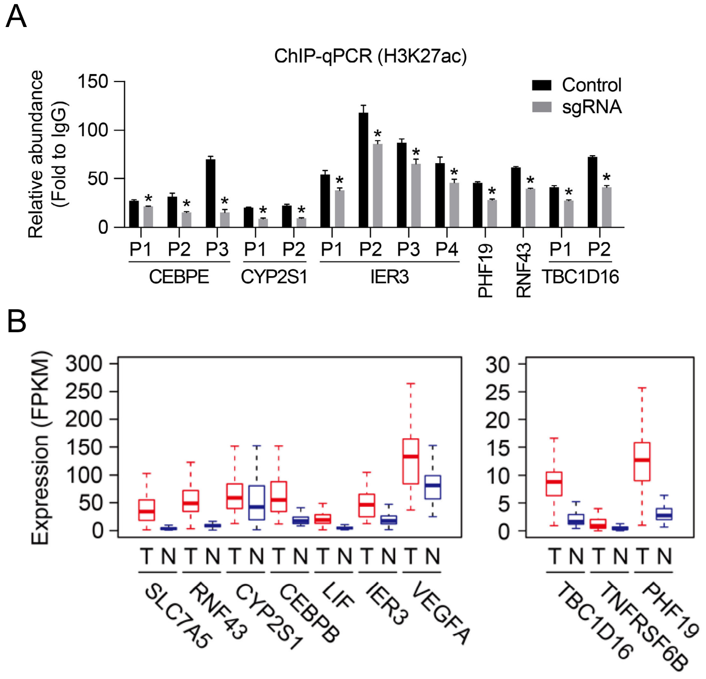

Sup. Fig. S10 ChIP-qPCR of super enhancers and expression of their corresponding genes in CRC. (A) ChIP-qPCR showing the H3K27ac level at CEBPE, CYP2S1, IER3, PHF19, RNF43 and TBC1D16 enhancer loci in control and sgRNA group. N = 3 for all groups, Data are presented as mean values +/- SEM. **(B)** Box plot for gene expression (FPKM) of 10 VSEL associated genes (*SLC7A5, RNF43, CYP2S1, CEBPB, LIF, IER3, VEGFA, TBC1D16, TNFRSF6B* and *PHF19*) in both tumor and native tissues. The box represent the data from 25th to 75th percentile, black line represent the median, and the top and bottom whiskers represent the 75th percentile + 1.5* interquartile range and 25th percentile + 1.5* interquartile range respectively. Statistical analysis was performed using an two-sided Student t test. * means p value < 0.05, ** means p value < 0.01, *** means p value < 0.001.

Sup. Fig. S11

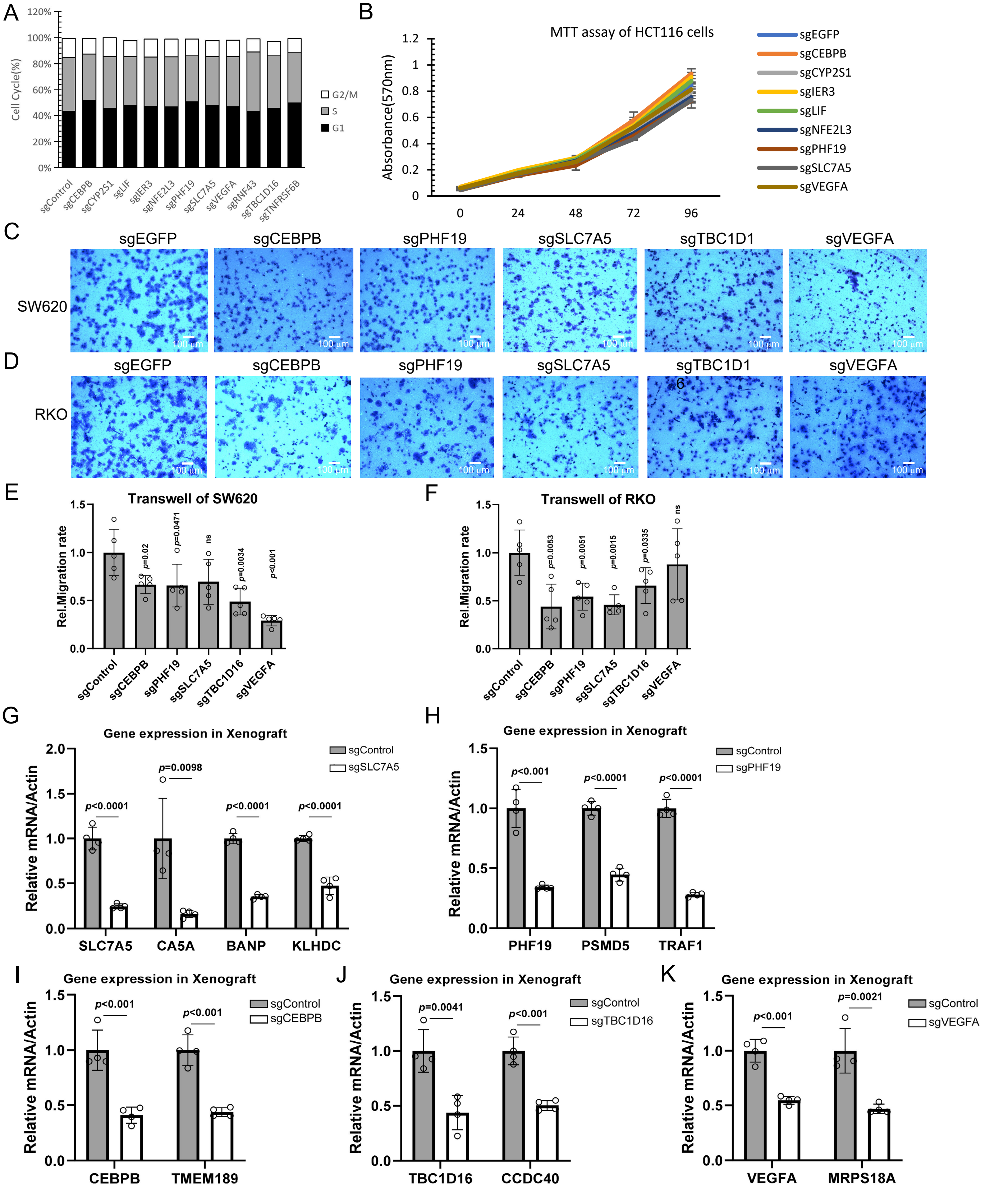

Sup. Fig. S11 Functional investigation of SEs in multiple CRC cell lines. (A) Cell cycle distributions analyzed by flowcytometry of stable cell lines with stable expression of the indicated CRISPR/sgRNAs. **(B)** Cell proliferation analyzed by MTT assay of the above cell lines. **(C&D)** Microscopic images of transwell assays performed with the indicated stable cell lines derived from SW620 or RKO cell lines. **(E&F)** Statistical analyses of C&D. n = 5. **(G-K)** Expression of the SE proximal genes in the xenograft tissues of Fig. 4G. n = 3. Data are presented as mean values +/- SEM. p vaule was labelled on the corresponding items.

Sup. Fig. S12

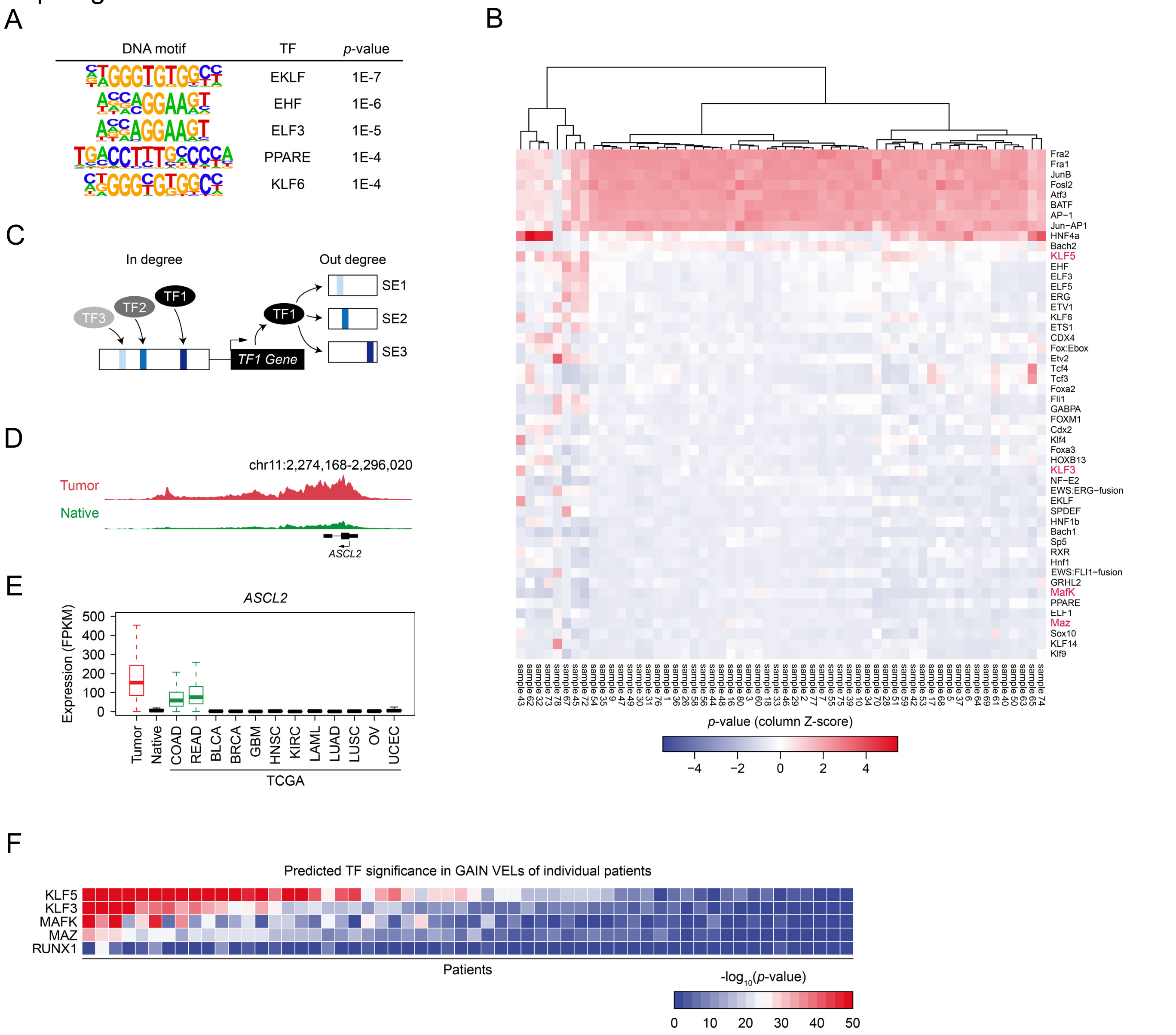

Sup. Fig. S12 Analysis of potential functional TFs in CRC. (A) DNA motifs enriched within nucleosome-free regions (NFRs) of lost VELs determined by HOMER motif analysis. P-value provided by HOMER. **(B)** Heatmap of top 50 transcription factors ranked by motif p-value calculated from gain VEL NFRs of CRC patients. P-value provided by HOMER. **(C)** Methodology for inferring the degree of core regulatory circuitry. **(D)** Meta normalized H3K27ac tracks at ASCL2 gene loci. **(E)** ASCL2 expression (FPKM) in patients of 12 cancer types. The group of our CRC tumor data was highlighted in red, and two intestinal cancer datasets from TCGA were highlighted in green. The box represent the data from 25th to 75th percentile, black line represent the median, and the top and bottom whiskers represent the 75th percentile + 1.5* interquartile range and 25th percentile + 1.5* interquartile range respectively. For the patient number of dataset, Tumor = 72, Native = 72, COAD = 657, READ = 166, BLCA = 412, BRCA = 1094, GBM = 166, HNSC = 501, KIRC = 484, LAML = 123, LUAD = 530, LUSC = 496, OV = 379, UCEC = 545.**(F)** Heatmap showing the significance (p-value) of transcription factors *KLF5*, *KLF3*, *MAFK*, *MAZ* and *RUNX1* in all patients. P-value provided by HOMER.

Sup. Fig. S13

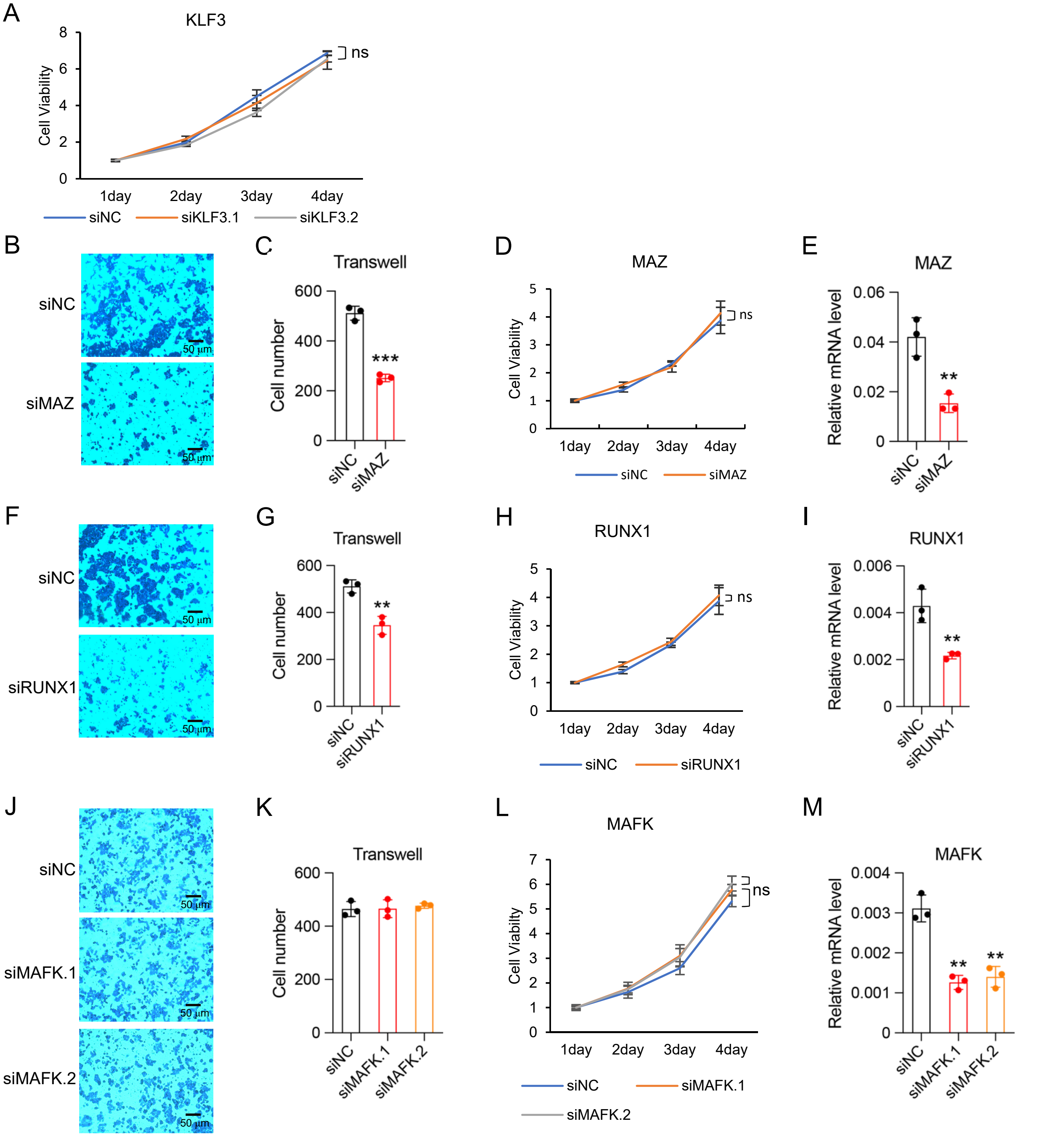

Sup. Fig. S13 Functional verification of predicted transcription factors . (A) Cell survival analysis of KLF3 knockdown cells. N = 3 independent experiments. Error bar represents ±SD. **(B-M)** Transwell and cell survival analysis of HCT116 cell with siRNAs of MAZ (B-E), RUNX1 (F-I), MAFK (J-M). N = 3 independent experiments. Data are presented as mean values +/- SEM. Statistical analysis was performed using an unpaired Student t test. For C, E, G and I, the p-value are 1.41E-4, 5.76E-3, 3.79E-3 and 7.21E-3; for M, p-value of siMAFK.1 = 1.06E-3, siMAFK.2 = 2.21E-3. ** means $p < 0.01$; *** means $p < 0.001$; ns means not significant.

Sup. Fig. S14

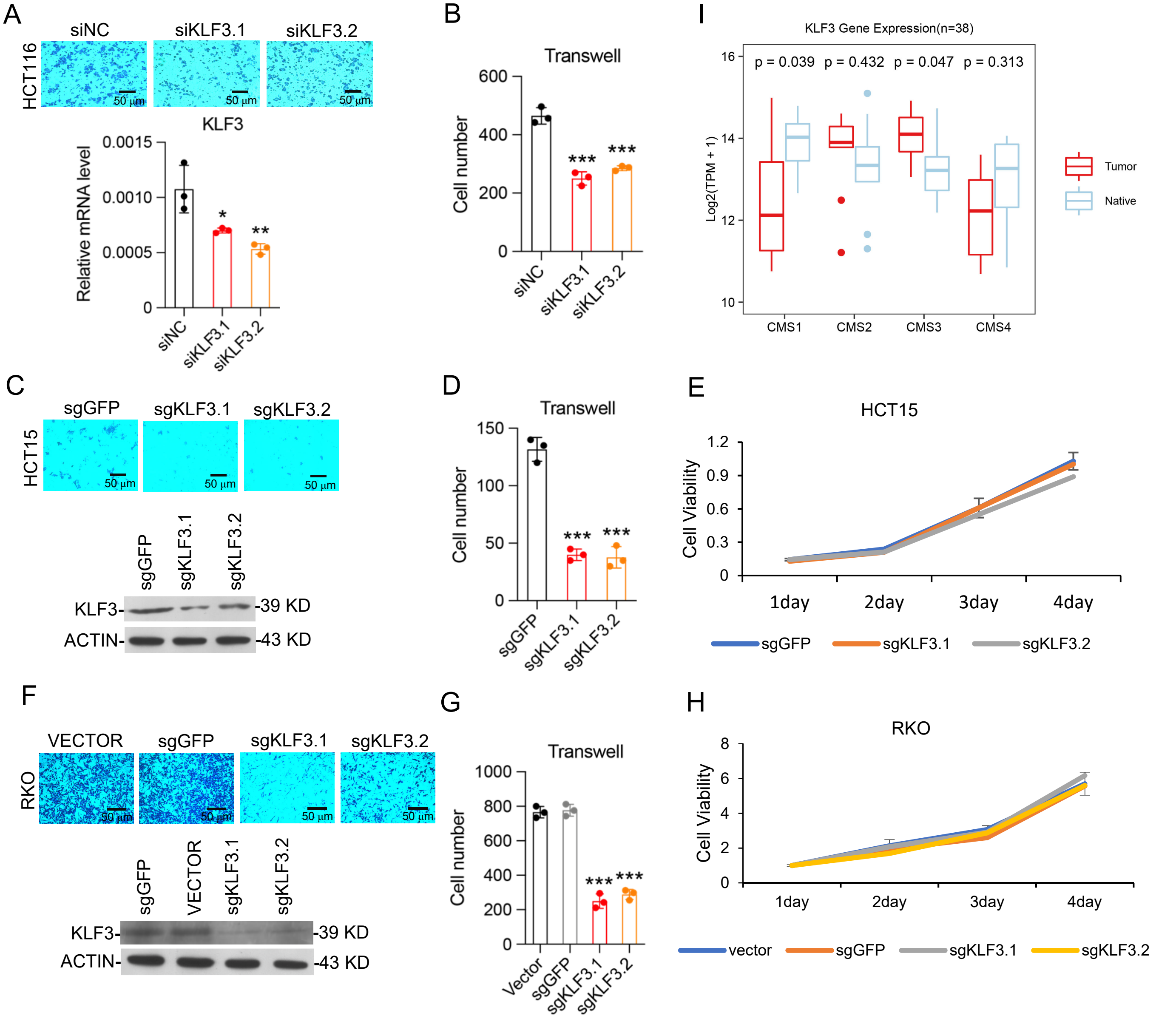

Sup. Fig. S14 KLF3 promotes CRC tumorigenesis. (A&B) *KLF3* was knocked down in HCT116 by two different siRNAs and transwell assay was performed. *KLF3* expression was measured with quantitative PCR. Cell migration was imaged and statistically analyzed. **(C-E)** *KLF3* knockdown by two different sgRNAs in HCT15 cell line. KLF3 was measured with western blotting. Cell migration was studied with transwell assay. The results was imaged and statistically analyzed. Cell proliferation was studied with MTT assay. For A, p-value of siKLF3.1 = 0.0402, siKLF3.2 = 0.0132; For B, p-value of siKLF3.1 = 5.07E-4, siKLF3.2 = 4.44E-4. N = 3 independent experiments. For D, p-value of siKLF3.1 = 1.62e-4, siKLF3.2 = 3.08E-4. Error bar represents ±SD. **(F-H)** *KLF3* knockdown by two different sgRNAs in RKO cell line. KLF3 was measured with western blotting. Cell migration was studied with transwell assay. The results was imaged and statistically analyzed. Cell proliferation was studied with MTT assay. For G, p-value of sgKLF3.1 = 7.47E-5, sgKLF3.2 = 4.75E-5. N = 3 independent experiments. Error bar represents ±SD. **(I)** Expression of KLF3 in four CMS subgroups. The results in all experiments represent the means (±SD) of at least three independent experiments. Statistical analysis was performed using an two-sided Student *t* test. ∗ p-value ≤ 0.05, ∗∗ p-value ≤ 0.01, ∗∗∗ p-value ≤ 0.001.

Sup. Fig. S15

A

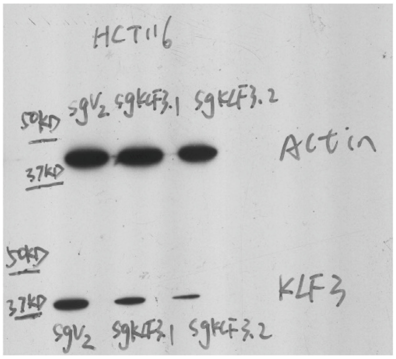

B

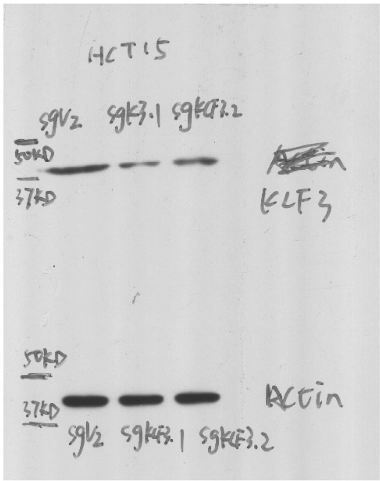

C

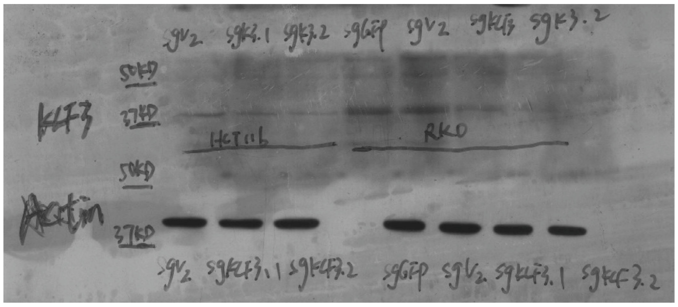

Sup. Fig. S15 Original films for western blots. (A) The original film for result of Fig. 5D. **(B)** The original film for result of Sup. Fig. S14C. **(C)** The original film for result of Sup. Fig. S14F.
