# Supplementary material for: Genome-wide profiling in colorectal cancer identifies PHF19 and TBC1D16 as oncogenic super enhancers
Source: Nat Commun. 2021 Nov 4;12:6407. doi: 10.1038/s41467-021-26600-5 (PMC8568941; doi:10.1038/s41467-021-26600-5)
Supplement: Supplementary file 4 — Reporting Summary [file 41467_2021_26600_MOESM4_ESM.pdf]

nature research

Corresponding author(s): Min Wu

Last updated by author(s): Oct 26, 2020

Reporting Summary

Nature Research wishes to improve the reproducibility of the work that we publish. This form provides structure for consistency and transparency in reporting. For further information on Nature Research policies, see our Editorial Policies and the Editorial Policy Checklist.

Statistics

For all statistical analyses, confirm that the following items are present in the figure legend, table legend, main text, or Methods section.

n/a	Confirmed	
☐	☒	The exact sample size (*n*) for each experimental group/condition, given as a discrete number and unit of measurement
☐	☒	A statement on whether measurements were taken from distinct samples or whether the same sample was measured repeatedly
☐	☒	The statistical test(s) used AND whether they are one- or two-sided<br>*Only common tests should be described solely by name; describe more complex techniques in the Methods section.*
☐	☒	A description of all covariates tested
☒	☐	A description of any assumptions or corrections, such as tests of normality and adjustment for multiple comparisons
☐	☒	A full description of the statistical parameters including central tendency (e.g. means) or other basic estimates (e.g. regression coefficient) AND variation (e.g. standard deviation) or associated estimates of uncertainty (e.g. confidence intervals)
☐	☒	For null hypothesis testing, the test statistic (e.g. *F*, *t*, *r*) with confidence intervals, effect sizes, degrees of freedom and *P* value noted<br>*Give P values as exact values whenever suitable.*
☒	☐	For Bayesian analysis, information on the choice of priors and Markov chain Monte Carlo settings
☒	☐	For hierarchical and complex designs, identification of the appropriate level for tests and full reporting of outcomes
☐	☒	Estimates of effect sizes (e.g. Cohen's *d*, Pearson's *r*), indicating how they were calculated

Our web collection on statistics for biologists contains articles on many of the points above.

Software and code

Policy information about availability of computer code

Data collection	Public ChIP-seq data from GEO database were collected by the software fastq-dump.2 (v 2.8.2), and the cancer patient RNA-seq data from TCGA were collected by gdc-client (v 1.6.0).
Data analysis	ChIP-seq data were analyzed by the software fastq-dump.2 (v 2.8.2), cutadapt (v 1.16), BWA (v 0.7.15), samtools (v 1.4.1), macs2 (v 2.1.1), HOMER (v 4.11), HTSeq (v 0.9.1), featureCounts (v 2.0.2), ROSE2, FactoMineR (v 2.3), GREAT website (v 3.0.0), CMScaller (v 0.99.2), Cytoscape (v.3.8.0), PARE (v 0.08) and crc software (https://github.com/linlabcode/CRC). The RNA-seq data for colorectal cancer patients were analyzed by cutadapt (v 1.16), hisat2 (v 2.1.0) and R package DESeq2 (1.26.0). The RNA-seq data from TCGA were pretreated and we did not use any software to analyze these data.

For manuscripts utilizing custom algorithms or software that are central to the research but not yet described in published literature, software must be made available to editors and reviewers. We strongly encourage code deposition in a community repository (e.g. GitHub). See the Nature Research guidelines for submitting code & software for further information.

Data

Policy information about availability of data

All manuscripts must include a data availability statement. This statement should provide the following information, where applicable:
- Accession codes, unique identifiers, or web links for publicly available datasets
- A list of figures that have associated raw data
- A description of any restrictions on data availability

We have removed the restriction to our CRC patient data in GEO. The statement of Data availability has been changed as below:
The raw data used in this study are available in the GEO database under accession code GSE156614 and GSE156613. The processed data are available in the supplemental tables.

20 colorectal cancer cell lines H3K27ac ChIP-seq data: GEO dataset - GSE77737. HCT116 BRD4 ChIP-seq data: GEO dataset - GSE126221. HCT116 H3K4me1 ChIP-seq data: ENCODE – ENCFF557VIT. Public cancer patient RNA-seq data were downloaded from TCGA database (TCGA-COAD, TCGA-READ, TCGA-BLCA, TCGA-BRCA, TCGA-GBM, TCGA-HNSC, TCGA-KIRC, TCGA-LAML, TCGA-LUAD, TCGA-LUSC, TCGA-OV and TCGA-UCEC).

Field-specific reporting

Please select the one below that is the best fit for your research. If you are not sure, read the appropriate sections before making your selection.

☒ Life sciences ☐ Behavioural & social sciences ☐ Ecological, evolutionary & environmental sciences

For a reference copy of the document with all sections, see nature.com/documents/nr-reporting-summary-flat.pdf

Life sciences study design

All studies must disclose on these points even when the disclosure is negative.

Sample size	We used the data from 73 pairs of CRC patient tissues (tumor tissue and adjacent native tissue) for this study. According to our saturation analysis, we found gained enhancers in tumor reached 80% when using less than 40 pairs of samples for analysis, and 90% with around 50 pairs, indicating the sample size used in our study was good enough for statistical analysis. For cell-based experiments, we used 3 biological replicates, which is widely accepted in the field.
Data exclusions	We collected 80 pairs of colorectal cancer patient tissues, but 7 pairs were unqualified because of the incorrect storage conditions, which were then excluded from the experiment. Considering the quality control results, we also excluded 9 pairs data in the analysis process. These 9 samples consist of two parts. 4 samples (patient #20, #21, #22, #24) were deleted because of their low number of significant H3K27ac peaks. Based on our experience and some published papers, we set 2500 as the number threshold, and we could see that the peak numbers of these 4 samples are less than 2500, which was may due to low quality of ChIP assay during sample preparation. We checked our H3K27ac ChIP-seq results for the 4 samples, including the concentration of the ChIP DNA and the sequencing reads number. We found the prepared samples of native tissues of the 4 pairs had less DNA than others, which was probably due to less cell number than other tissues, which is often a nightmare for ChIP assay with patient tissues. Their paired tumor data is normal, and if we keep these 4 patients for VEL identifying, we'll get lots of false candidates, which is not good for our analysis. These criteria were pre-established.
Replication	We performed at least 3 biological replications for all experimental verification, and the results shown were reproducible.
Randomization	The patients were allocated into this study randomly. We select the patients who performed colorectal cancer surgury in the Zhongnan hospital regardless of their gender, age, malignancy grade of tumor and the time point they went to the hospital.
Blinding	We were not blinded to the patient information. We choose the patients randomly but we need their information (like gender and age) to research the clinical relevance in our study. And in our study, this blinding will not affect our conclusion in patient enhancer related analysis. For other experiments, the investigators were not blinded to group allocation during data collection and analysis, because they prepared and performed the experiments by themselves, and no other persons were involved. The corresponding authors oversaw the design and data analysis of each experiment to ensured the accuracy of the results.

Reporting for specific materials, systems and methods

We require information from authors about some types of materials, experimental systems and methods used in many studies. Here, indicate whether each material, system or method listed is relevant to your study. If you are not sure if a list item applies to your research, read the appropriate section before selecting a response.

Materials & experimental systems

n/a	Involved in the study	
☐	☒	Antibodies
☐	☒	Eukaryotic cell lines
☒	☐	Palaeontology and archaeology
☐	☒	Animals and other organisms
☐	☒	Human research participants
☒	☐	Clinical data
☒	☐	Dual use research of concern

Methods

n/a	Involved in the study	
☐	☒	ChIP-seq
☒	☐	Flow cytometry
☒	☐	MRI-based neuroimaging

Antibodies

Antibodies used	H3K4me3, Millipore (Merck), 04-745, 2965110, no dilution for ChIP-seq; H3K27ac, Abcam, ab4729, GR3216173-1, no dilution for ChIP-seq; KLF3, Abclonal A7195, RRID:AB_2767745, 1:1,000 for western blot.
Validation	The validation of H3K4me3 in our lab was performed in the previous study (Li Q.L., et al. The hyper-activation of transcriptional enhancers in breast cancer. Clin Epigenetics 2019, 11:48.). The merchant validation can be found at the following link, https://www.merckmillipore.com/CN/zh/product/Anti-trimethyl-Histone-H3-Lys4-Antibody-clone-MC315-rabbit-

monoclonal,MM_NF-04-745.
For H3K27ac antibody, the correlations between the ChIP-Seq results with the online data about BRD4 and H3K4me1 were calculated (Sup. Fig. S2B&C). The merchant validation can be found at the following link, https://www.abcam.com/histone-h3-acetyl-k27-antibody-chip-grade-ab4729.html.
For KLF3 antibody, the merchant validation can be found at the following link, https://abclonal.com.cn/catalog/A7195. In the current study, we validated it with sgRNA and siRNA knockdown assays (Fig. 5D, Sup. Fig. S14C & F).

Eukaryotic cell lines

Policy information about cell lines

Cell line source(s)
HCT116, HCT15 and RKO Cell lines were purchased from Cell Bank of Chinese Academy.

Authentication
Authentication was performed by Cell Bank of Chinese Academy, and we did not check again after we got it.

Mycoplasma contamination
Mycoplasma was tested negative by Cell Bank of Chinese Academy, and we did not check again after we got it.

Commonly misidentified lines
(See ICLAC register)
None

Animals and other organisms

Policy information about studies involving animals; ARRIVE guidelines recommended for reporting animal research

Laboratory animals
For laboratory animals, report species, strain, sex and age OR state that the study did not involve laboratory animals.

Wild animals
Provide details on animals observed in or captured in the field; report species, sex and age where possible. Describe how animals were caught and transported and what happened to captive animals after the study (if killed, explain why and describe method; if released, say where and when) OR state that the study did not involve wild animals.

Field-collected samples
For laboratory work with field-collected samples, describe all relevant parameters such as housing, maintenance, temperature, photoperiod and end-of-experiment protocol OR state that the study did not involve samples collected from the field.

Ethics oversight
All the animal operations were following the laboratory animal guidelines of Wuhan University and were approved by the Animal Experimentations Ethics Committee of Wuhan University (Protocol NO. 14110B).

Note that full information on the approval of the study protocol must also be provided in the manuscript.

Human research participants

Policy information about studies involving human research participants

Population characteristics
Describe the covariate-relevant population characteristics of the human research participants (e.g. age, gender, genotypic information, past and current diagnosis and treatment categories). If you filled out the behavioural & social sciences study design questions and have nothing to add here, write "See above."

Recruitment
Describe how participants were recruited. Outline any potential self-selection bias or other biases that may be present and how these are likely to impact results.

Ethics oversight
Identify the organization(s) that approved the study protocol.

Note that full information on the approval of the study protocol must also be provided in the manuscript.

ChIP-seq

Data deposition

☒ Confirm that both raw and final processed data have been deposited in a public database such as GEO.

☒ Confirm that you have deposited or provided access to graph files (e.g. BED files) for the called peaks.

Data access links
May remain private before publication.
https://www.ncbi.nlm.nih.gov/geo/query/acc.cgi?acc=GSE156613
Secure token: qxejeiuqhjivdgt

Files in database submission
All files in GEO dataset GSE156613 are the ChIP-seq files for this study.

Genome browser session
(e.g. UCSC)
All bigwig files for ChIP-seq data have been deposited to GEO dataset GSE156613 as processed file.

Methodology

Replicates
73 pairs of tissues were used for ChIP-Seq. For experiments in cells, at least three biological replicates were performed.

Sequencing depth	The information was provided in Extended Data Table 2.
Antibodies	H3K4me3, Millipore, EMD-04-745, 2965110; H3K27ac, Abcam, ab4729, GR3216173-1.
Peak calling parameters	Peaks calling was finished by MACS2 (v 2.1.1) with the parameters --nomodel --keep-dup all -p 1E-10 --broad --broad-cutoff 1E-10 --extsize 147.
Data quality	FASTQC v0.11.8 is run to check the sequencing quality.
Software	FASTQC 0.11.8; cutadapt (v1.16); BWA (v 0.7.15); samtools (v1.4.1); macs2 (v2.1.1). The detailed description have been provided in Method.
